# Status quo and influencing factors of readiness for hospital discharge in patients with brain tumours after surgery

**DOI:** 10.3389/fonc.2024.1324810

**Published:** 2024-09-12

**Authors:** Yue-Hong Qin, Xiao-Mei Shi

**Affiliations:** Department of Neurosurgery, Shandong Provincial Hospital Affiliated to Shandong First Medical University, Jinan, Shandong, China

**Keywords:** brain tumours, readiness for discharge, current situation, discharge preparation measurement table, discharge guidance quality scale

## Abstract

**Objective:**

This study aimed to investigate the status quo of readiness for hospital discharge in patients with brain tumours after surgery and to analyse its influencing factors.

**Method:**

A total of 300 patients with brain tumours who were admitted to the neurosurgery ward of our hospital between September 2020 and December 2022 were selected as the study participants using the convenient sampling method. The readiness for hospital discharge in patients with brain tumours after surgery was investigated using a general information questionnaire, the Readiness for Hospital Discharge Scale (RHDS), the Quality of Discharge Teaching Scale (QDTS), the University of Washington Quality of Life Questionnaire (UW-QOL), and the Social Support Rating Scale (SSRS), and its influencing factors were analysed.

**Results:**

The total RHDS score of patients with brain tumours was (155.02 ± 14.67), which was at a medium level. There was a positive correlation between readiness for hospital discharge in patients with brain tumours after surgery and the UW-QOL score (*r* = 0.459, *p* = 0.001), SSRS score (*r* = 0.322, *p* = 0.000), and QDTS score (*r* = 0.407, *p* = 0.001). The influencing factors of readiness for hospital discharge in patients with brain tumours included the content actually obtained by patients (health guidance) before discharge (*p* = 0.001), discharge teaching skills (*p* = 0.001), age (*p* = 0.006), swallowing status (*p* = 0.021), education level (*p* = 0.016), and objective support (*p* = 0.022).

**Conclusion:**

The readiness for hospital discharge in patients with brain tumours is at a medium level. Medical staff should give inpatients more targeted knowledge and implement personalised health education according to the patient’s age, education level, swallowing status, and objective support to improve the patient’s readiness for hospital discharge.

## 1 Introduction

Brain tumours are an important factor in the global cancer burden. The latest data show that there are approximately 900,000 new cases and 400,000 deaths in the world every year (1), and the incidence rate ranks seventh among malignant tumours (2).

Brain tumours refer to intracranial nervous system tumours that exhibit expansive and infiltrative growth, causing compression of brain tissue and seriously affecting nervous system function (3). Brain tumours are one of the common tumours in neurosurgery, and surgery is a commonly used treatment method with good clinical results (4). Surgical removal of all or most of the tumour can reduce the space-occupying effect, thereby relieving or alleviating the compression of the tumour on the functional parts of the brain tissue and eliminating or reducing adverse effects (5). However, some patients experience negative psychological effects due to long-term compression damage to the brain tissue caused by the tumour, surgical operation damage, or other reasons (6). Advances in treatment have prolonged life expectancy in neuro-oncological patients, and the long-term preservation of their quality of life is, therefore, a new challenge (7). Patients often experience functional impairments, such as in language, chewing, swallowing, and sleep, after surgery and these may even be accompanied by problems such as limited mobility, dysarthria, and limited ventilation (8). Therefore, before discharge, patients should master the early identification, prevention, and corresponding functional exercise methods for postoperative complications of brain tumours. Adequate discharge preparation is a key step in the rehabilitation process of patients with brain tumours.

Readiness for hospital discharge refers to the comprehensive assessment of a patient’s physiological, psychological, and social status by medical personnel to determine whether they are capable of leaving the hospital, returning to society, and receiving further rehabilitation (9). Existing studies have shown that the rate of unplanned readmission, the incidence of complications, the mortality rate, and the economic burden of patients can be reduced if adequate preparation for hospital discharge is carried out (10, 11). The studies, both at home and abroad, show that readiness for hospital discharge in patients is closely related mainly to social support, the quality of discharge guidance, disease status, personal characteristics, and quality of life (12–15). The sex, marital status, treatment period, quality of discharge teaching, and fear of disease progression influenced discharge readiness among patients receiving chemotherapy for lung cancer (16). The readiness for hospital discharge of patients with brain tumours may be influenced by some of these factors, and exploring them may play an important role in making improvements. In this study, the current situation of readiness for hospital discharge in patients with brain tumours in Shandong Provincial Hospital Affiliated with Shandong First Medical University was investigated. Its influencing factors were analysed to improve the readiness for hospital discharge in patients with brain tumours after surgery.

## 2 Materials and methods

### 2.1 Research participants

A total of 300 patients with brain tumours who were admitted to the Shandong Provincial Hospital Affiliated with Shandong First Medical University between September 2020 and December 2022 were included in this study using convenience sampling. In general, a study’s sample size should be 10–15 times that of the survey items. In this study, the Quality of Discharge Teaching Scale (QDTS) consisted of 24 entries; therefore, the sample size should be 240–360 cases. Considering the patient numbers in the hospital, the sample size of this study was chosen to be 300 patients. The inclusion criteria were as follows: the patient (1) was diagnosed with a brain tumour and received surgical treatment; (2) was aged ≥18 years; (3) was pathologically diagnosed with a malignant tumour; (4) had no previous mental disorder; (5) had been aware of the disease diagnosis, had given informed consent to this study, and voluntarily participated in it; (6) was hospitalised for >3 d; (7) was intended to be discharged from hospital on the day of the investigation. The exclusion criteria were as follows: the patient had (1) cognitive impairment; (2) abnormal function of other organs (such as heart, lung, liver, or kidney). This study has been approved by the Ethics Committee of the hospital.

### 2.2 Investigation tools

(1) General information questionnaire: the questionnaire was designed by the researcher based on a literature search (17). It had 16 items, including age, gender, education level, occupation, marital status, lifestyle, place of residence, monthly income of the family, payment method for medical expenses, location of disease, duration of disease, pathological classification, surgical method, pain score at discharge (using the digital scoring method), length of hospitalisation, and whether a gastric tube was inserted at discharge. Most of these data were queried and exported from the hospital information system, and a small amount of information, such as monthly income, was obtained by researchers directly asking the patients.

(2) Readiness for Hospital Discharge Scale (RHDS) (18): The scale contains a total of 23 items in four dimensions, including physical state (7 items), out-of-hospital coping ability (3 items), disease knowledge (8 items), expected support (4 items), and whether the patient is ready for hospital discharge (a single item). Among the above items, the single item is a yes or no question, and the four-dimensional items are degree scores. Items 2 and 5 are scored in reverse, and the remaining items are scored 0–10 points from ‘not ready’ to ‘fully ready’, with a total score of 0–220 points. The higher the score is, the better the readiness for hospital discharge. The overall Cronbach’s alpha coefficient of this scale in this study was 0.91, and the value for each dimension was 0.73–0.89, which had good reliability and validity.

(3) Quality of Discharge Teaching Scale (19): The scale contains a total of 24 items in three dimensions: the content that patients need to obtain before discharge [6 items (not included in the total score)], the content actually obtained by patients before discharge (6 items), and discharge teaching skills (12 items). The scoring method of 0–10 points is adapted to each item, and the total score is 0–180 points. Participants evaluated whether they had received good care and sufficient medical information; for example, whether the nurse’s guidance was timely, whether the nurse’s knowledge was helpful, and whether the participants had received the necessary medical information and exercises. The higher the score is, the higher the quality of discharge guidance. The overall Cronbach’s alpha coefficient of this scale in this study was 0.872, and the value for each dimension was 0.69–0.83, which had good reliability and validity.

(4) University of Washington Quality of Life Questionnaire (UW-QOL) (20): The Chinese-language version of UW-QOL consists of 12 symptom items, such as pain, appearance, chewing, speaking, and swallowing, with a total score of 0–120. Participants conducted self-assessment according to the above items and chose the appropriate option; for example, whether there was pain; whether there were obstacles in swallowing, speaking, or chewing; and whether they felt anxiety. The higher the score is, the better the quality of life. The Cronbach’s alpha coefficient of this scale in this study was 0.816.

(5) Social Support Rating Scale (SSRS) (21): The scale contains a total of 10 items in three dimensions, including objective support (3 items), subjective support (4 items), and utilisation of support (3 items). The total score is 12–66 points. Participants evaluated their interpersonal relationships, including ways of interacting with colleagues, neighbours, and friends, as well as when seeking help. The higher the score is, the more the overall support. The Cronbach’s alpha coefficient of this scale in this study was 0.854.

### 2.3 Methods of data collection

**Figure 1:**
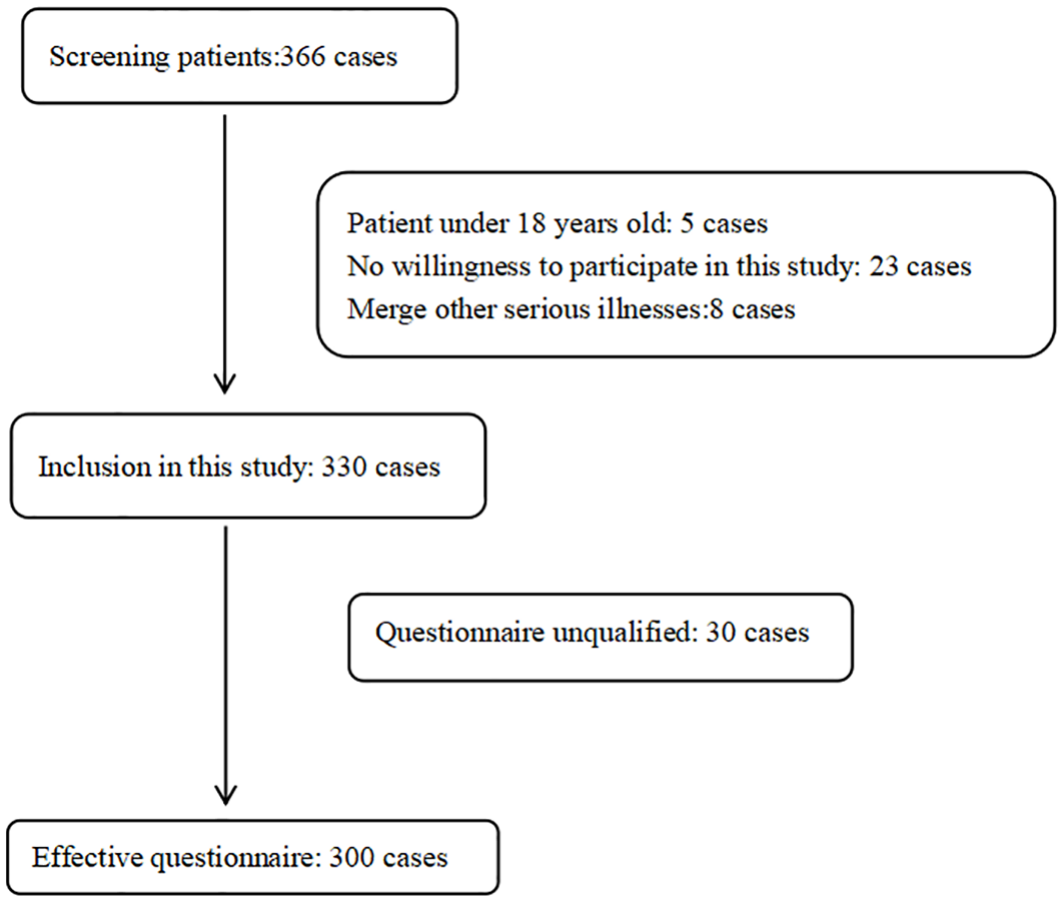
The screening process for participants in this study.

Nurses with uniform training and more than 5 years of working experience in a neurosurgery ward were selected as investigators. The questionnaires were distributed to the patients on-site on the day of their discharge after obtaining their consent. The investigators used unified guidelines to state the research purpose and filling-in method to the patients, and the questionnaire was filled in by the patient according to the actual situation. The questionnaires were checked by the investigators after they were completed, and the respondents were asked to complete the questionnaire if there were any missing items. When the questionnaires were completed, they were immediately taken back. A total of 330 questionnaires were sent out, and 300 were effectively collected, with an effective recovery rate of 91.00%. The screening process for participants in this study is shown in **Figure 1**.

### 2.4 Statistical methods

The data were input using Excel and checked by two people. SPSS 25.0 software was used for statistical analysis. Qualitative data were described by frequency and percentage (%). Quantitative data satisfying normal distribution were described by mean ± standard deviation ( $\bar{\text{x}}$ ± s) and compared between groups by independent sample *t*-test and ANOVA. Quantitative data that did not satisfy the normal distribution were described as the median (interquartile distance) (M[QR]) and compared between groups using the Kruskal-Wallis test. The basic data of the patients met a normal distribution, and the overall variance was homogeneous in this study. Pearson correlation was used to analyse the degree of correlation among the variables. Multiple linear regression was used to analyse the influencing factors of readiness for hospital discharge in patients after brain tumour surgery. Results were considered statistically significant if *p <*0.05.

## 3 Results

### 3.1 General information on postoperative patients with brain tumours

The age of the 300 patients with brain tumours ranged from 19 to 82 (54.39 ± 12.49) years. There were 180 men (60%) and 120 women (40%). There were 92 patients (30.67%) with a college education. Most of the patients were married (262 patients, 87.33%). Most cases were meningioma (223 patients, 74.33%).

### 3.2 Status quo of readiness for hospital discharge in patients after brain tumour surgery

The total RHDS score in patients with brain tumours was 155.02 ± 14.67, which was at the middle level. The score of the body status dimension was 55.47 ± 4.92, and the average score of the items was 8.48 ± 0.73 points. The score of the disease knowledge dimension was 55.35 ± 6.51, and the average score of the items was 6.52 ± 0.92. The score of the coping ability dimension outside the hospital was 19.31 ± 3.82, and the average score of the items was 7.53 ± 0.86. The score of the expected support dimension was 27.23 ± 6.18, and the average score of the items was 8.51 ± 1.06.

### 3.3 Univariate analysis of readiness for hospital discharge in patients after brain tumour surgery

The analysis showed that age, monthly income of the family, education level, marital status, whether a gastric tube was necessary at discharge, and pathological classification were the influencing factors of readiness for hospital discharge in the patients after brain tumour surgery (*p* < 0.05). See **Table 1**.

**Table 1:** Comparison of discharge readiness of patients with different characteristics of brain tumours after surgery (n=300).

project	Classification	Number of cases (%)	RHDS total score $\left(\bar{\text{x}}\pm \text{sd}\right)$	Statistical value	*P*
Age (years)	≦40	31 (10.33)	159.91 ± 18.03	F=11.602	0.001
	40~49	36 (12.00)	160.90 ± 13.89		
	50~59	49 (16.33)	160.23 ± 12.38		
	60~69	130 (43.33)	154.10 ± 10.36		
	≧70	54 (18.01)	147.06 ± 12.50		
Family monthly income (yuan)	≤2000	112 (37.33)	153.21 ± 10.64	F=4.205	0.031
	2001~3000	35 (11.67)	155.32 ± 14.26		
	3 001~4 000	31 (10.33)	150.67 ± 19.42		
	4001~5000	36 (12.00)	161.20 ± 15.23		
	>5000	86 (28.67)	163.48 ± 15.61		
Education level	Primary school and below	40 (13.33)	145.08 ± 13.62	F=5.002	0.003
	junior high school	48 (16.00)	153.51 ± 11.67		
	High school or technical secondary school	77 (25.67)	159.94 ± 12.48		
	Junior college	92 (30.67)	161.80 ± 13.58		
	Undergraduate	43 (14.33)	164.02 ± 11.66		
marital status	married	262 (87.33)	157.27 ± 11.51	F=5.672	0.001
	unmarried	12 (4.00)	164.67 ± 17.36		
	Divorce	7 (2.33)	161.47 ± 7.60		
	Widow	19 (6.34)	142.81 ± 13.75		
Is a gastric tube retained upon discharge	yes	64 (21.33)	150.55 ± 17.38	t=-3.561	0.002
	no	236 (78.67)	16453 ± 16.87		
Pathological classification	meningioma	223 (74.33)	151.56 ± 13.51	F=5.041	0.036
	Glioma	40 (13.33)	163.40 ± 13.69		
	Metastatic tumour	33 (11.00)	165.19 ± 10.94		
	other	4 (1.34)	162.54 ± 16.31		

RHDS, Hospital Discharge Scale; $\left(\bar{\text{x}}\pm \text{sd}\right)$ , mean ± standard deviation.

### 3.4 Correlation between readiness for hospital discharge and quality of life, social support, and discharge guidance in patients after brain tumour surgery

The Pearson correlation analysis results showed that the readiness for hospital discharge in the patients after brain tumour surgery was positively correlated with the UW-QOL scores (*r* = 0.459, *p* = 0.001), SSRS scores (*r* = 0.322, *p* < 0.001), and QDTS scores (*r* = 0.407, *p* = 0.001). The results are shown in **Table 2**.

**Table 2:** Correlation analysis of discharge readiness, quality of life, social support, and quality of discharge guidance in patients with brain tumours after surgery (n=99).

Project	r value	*p*
SSRS total score	0.322	0.000
Subjective support	0.057	0.121
Objective support	0.405	0.002
Utilisation of support	0.157	0.005
QDTS total score	0.407	0.001
What patients need to obtain before discharge	0.123	0.029
What the patient actually obtained before discharge	0.484	0.001
Discharge Guidance Techniques	0.512	0.001
UW-QOL total score	0.459	0.001
pain	0.301	0.001
appearance	0.402	0.001
activity	0.171	0.004
entertainment	0.083	0.139
swallow	0.348	0.001
chew	0.510	0.001
Gustatory sensation	0.205	0.002
saliva	0.023	0.452
language	0.340	0.001
Shoulder function	0.146	0.021
emotion	0.312	0.001
anxious	0.210	0.037

### 3.5 Analysis of influencing factors on readiness for hospital discharge in patients with brain tumours

Linear step-by-step regression analysis was performed by comparing the readiness for hospital discharge in the patients with different characteristics of brain tumours after surgery. The statistically significant variables in the correlation analysis of discharge readiness, quality of life, social support, and discharge guidance quality of patients with brain tumours after surgery were used as independent variables, and the RHDS scores of patients with brain tumours were used as the dependent variables. The results showed that the content actually obtained by patients (health guidance) before discharge (*p* = 0.001), discharge teaching skills (*p* = 0.001), age (*p* = 0.006), swallowing status (*p* = 0.021), education level (*p* = 0.016), and objective support (*p* = 0.022) were the factors influencing the readiness for hospital discharge in patients with brain tumours. See **Table 3**.

**Table 3:** Multiple linear regression analysis of influencing factors on discharge readiness of brain tumour patients.

project	Regression coefficient	Standard error	Standardised regression coefficient	DW	T value	*P*
constant	40.104	1.498	–	1.92	26.362	0.001
What the patient actually obtained before discharge	2.142	0.037	1.500	2.13	68.003	0.001
Discharge Guidance Techniques	1.714	0.079	0.571	2.08	22.104	0.001
Age	-1.211	0.119	-0.078	1.86	9.001	0.006
swallow	0.037	0.007	0.059	2.35	6.231	0.021
Education level	1.104	0.324	0.084	2.21	5.305	0.016
Objective support	0.113	0.044	0.025	2.26	2.571	0.022

Durbin-Watson.

### 3.6 Multi-fold cross-validation and statistical testing

To verify the importance of the findings reported in this study, we further analysed the data using 10-fold cross-validation. The data from the 300 patients were randomly divided into 10 folds, with 9 folds used as the training set and the remaining 1 fold as the test set each time, repeating the experiment 10 times, and calculating the average result. The multi-fold cross-validation results showed that the main factors affecting patients’ readiness for discharge were still the content actually obtained (health guidance), discharge teaching skills, age, swallowing status, education level, and objective support, which was consistent with the previous linear regression analysis results.

To test the statistical significance of these influencing factors, we further conducted an ANOVA. The results showed that the effects of the content actually obtained (F=18.75, p<0.001), discharge teaching skills (F=21.27, p<0.001), age (F=7.08, p=0.009), swallowing status (F=5.44, p=0.022), education level (F=6.12, p=0.015), and objective support (F=5.96, p=0.017) on readiness for discharge were statistically significant.

## 4 Discussion

Patients’ physiology and psychology are seriously affected in the treatment of brain tumours. The incidence of complications may be increased when they are discharged from the hospital with a poor mental state, poor communication skills, and unmet anticipated support. Patient safety after discharge, adherence to treatment, and time to next admission may be affected by readiness for hospital discharge. Therefore, the core of this study was to evaluate the readiness for hospital discharge in patients after surgery for brain tumours and explore the influencing factors from the perspective of patients.

The results of this study showed that the total score of RHDS in patients with brain tumours after surgery was at a medium level, similar to the results of Huang XL et al. (22). They indicated that both the discharge plan of patients during hospitalisation and the coping ability outside of the hospital should be improved when the readiness for hospital discharge in patients with brain tumours was at a medium level. The average score of items in the dimension of disease knowledge was low, as shown by comparing the average score of RHDS items in each dimension. This indicated that patients’ medical understanding of brain tumours was insufficient, which may be due to the low incidence of brain tumours and limited efforts to educate the patients. This may indicate that health promotion before discharge is not sufficient. The nursing staff should attempt to improve disease knowledge education and strengthen the content of the education during the period of hospitalisation. They could, for example, rhetorically question the patients and their family members using scenario simulation to ensure that both the patients and their family members participate in the education work to improve the patient’s knowledge of the disease. In this study, the correlation between the RHDS and the QDTS, UW-QOL, and SSRS scores was moderate or low. This may be due to multiple factors that affect discharge preparation. The mixing of multiple factors affects the final result. Some studies have explored the correlation between RHDS and QDTS, and the results are similar to this study (16, 23). However, one study has shown that the quality of discharge instruction can reduce the risk of readmission and improve overall patient satisfaction (24). Therefore, we believe that the QDTS, UW-QOL, and SSRS have significant contributions to RHDS, although their correlation is not particularly strong.

The study showed that younger patients with brain tumours had higher RHDS scores, which was consistent with the results of Zhao et al. (25). The RHDS scores of patients aged <40 years with brain tumours were higher than those aged ≥40 years in this study. This may be related to more complications after general anaesthesia, physiological function decline, slow recovery of health status, lower overall education level, and poor understanding of disease expertise in elderly patients. In addition, the RHDS scores of the patients with brain tumours and better swallowing function were higher, which was similar to the results of Wen Zuozhen et al. (26). Therefore, patients with limited swallowing function should be informed in detail in clinical work about ways to improve swallowing function and ways to increase nutrient intake.

This study also showed that objective social support was an influential factor in the readiness for hospital discharge, and the RHDS score of patients with brain tumours was higher when the objective support was better, which was similar to the results of previous studies (27). Patients with a higher level of objective support have a stronger material foundation, which can be used as a protective factor in hospital discharge preparation and lay an economic foundation for further treatment. The patients’ sense of security can be increased, and their confidence in overcoming disease can be improved by receiving good social support; the patients will then pay more attention to and comply with disease knowledge. Therefore, clinical nursing staff should guide family members to pay attention to and participate in the whole process of the patient’s disease treatment according to the level of objective support to help the patient establish confidence in overcoming the disease, improve their readiness for hospital discharge, and enhance their adaptability after discharge.

The results showed that the content actually obtained by patients (health guidance) before discharge, discharge teaching skills, age, swallowing status, education level, and objective support were the factors influencing the readiness for hospital discharge in patients with brain tumours. This was similar to Wang et al.’s study (16) since they emphasised the importance of quality of discharge teaching; however, this study has identified more influencing factors, including age, swallowing status, education level, and objective support.

## 5 Limitations and strengths

This study investigated the current situation of readiness for hospital discharge in patients with brain tumours and analysed its influencing factors with the aim of improving it. However, the study has certain limitations, such as a limited sample size and the inclusion of patients solely from the surgery ward of the Neurosurgery department in a tertiary A hospital. This weakens the universality of the study results. Furthermore, this study used a questionnaire to collect data, and the subjective influence of participants may cause bias in the results. The reliance on self-reported and retrospective data may also introduce bias. In future studies, we will include more samples and use multiple centres. The follow-up qualitative research and longitudinal follow-up assessing post-discharge outcomes were necessary to gain deeper insights, thus providing practical guidelines for incorporating findings into discharge planning. The interdisciplinary collaborations for holistic discharge management were helpful and necessary for replication in diverse settings/populations.

## 6 Conclusion

The study results showed that the readiness for hospital discharge in patients with brain tumours was at a medium level. The influencing factors of the readiness for hospital discharge in these patients were the content that the patients actually obtained (health guidance) before discharge, discharge teaching skills, age, swallowing status, education level, and objective support. Nursing staff should focus on elderly patients with relatively low education levels, weak objective support, and poor swallowing function, and they should adopt personalised education methods, improve discharge teaching skills, and increase the knowledge that patients obtain in the hospital. This will improve the patients’ readiness for hospital discharge, promote their rehabilitation, and enable a successful return to society.

## Data Availability

The original contributions presented in the study are included in the article/**Supplementary Material**. Further inquiries can be directed to the corresponding author.
